# Pharmacodynamics of Rivaroxaban and Dabigatran in Adults with Diffuse Large B-Cell Lymphoma Receiving R-CHOP Immunochemotherapy

**DOI:** 10.3390/pharmaceutics16101319

**Published:** 2024-10-11

**Authors:** Teerachat Punnachet, Tim R. Cressey, Porntipa Apiwatnakorn, Atisa Koonarat, Lalita Norasetthada, Adisak Tantiworawit, Ekarat Rattarittamrong, Thanawat Rattanathammethee, Sasinee Hantrakool, Pokpong Piriyakhuntorn, Nonthakorn Hantrakun, Piangrawee Niprapan, Chatree Chai-Adisaksopha

**Affiliations:** 1 Division of Hematology, Department of Internal Medicine, Faculty of Medicine, Chiang Mai University, Chiang Mai 50200, Thailand; teerachat.pun@cmu.ac.th (T.P.); lalita.n@cmu.ac.th (L.N.); adisak.tan@cmu.ac.th (A.T.); ekarat.r@cmu.ac.th (E.R.); thanawat.r@cmu.ac.th (T.R.); sasinee.h@cmu.ac.th (S.H.); pokpong.p@cmu.ac.th (P.P.); nonthakorn.h@cmu.ac.th (N.H.); piangrawee.n@cmu.ac.th (P.N.); 2 AMS-PHPT Research Collaboration, Faculty of Associated Medical Sciences, Chiang Mai University, Chiang Mai 50200, Thailand; tim.cressey@phpt.org; 3 Department of Internal Medicine, Lamphun Hospital, Lamphun 51000, Thailand; a_nulek@windowslive.com; 4 Department of Internal Medicine, Nakornping Hospital, Chiang Mai 50180, Thailand; atisa.knrt@gmail.com

**Keywords:** rivaroxaban, dabigatran, R-CHOP, drug–drug interaction

## Abstract

**Background/Objectives**: Rivaroxaban and dabigatran are commonly used for thromboembolic disease management in active cancer patients. However, limited research explores the impact of concurrent chemotherapy on the pharmacodynamics of direct oral anticoagulants (DOAC). The aim of our study was to evaluate the impact of combined chemotherapy with rivaroxaban and dabigatran on the pharmacodynamics in patients with diffuse large B-cell lymphoma (DLBCL).; **Methods**: This was a prospective, pharmacodynamic study. Eligible subjects were ≥18 years old, diagnosed with DLBCL and initiating R-CHOP (rituximab, cyclophosphamide, doxorubicin, vincristine, and prednisone) immunochemotherapy. The enrolled adults received either rivaroxaban (10 mg once daily) or dabigatran etixalate (110 mg twice daily). Plasma anti-factor Xa (FXa) in participants on rivaroxaban and diluted thrombin time (dTT) in participants on dabigatran were assessed over the dosing interval before and after R-CHOP administration. Pharmacodynamic parameters of rivaroxaban and dabigatran were determined using a non-compartmental analysis.; **Results**: Twenty-six adults participated, with twelve in the rivaroxaban group and fourteen in the dabigatran group. The mean age was 59 ± 14.4 years. In the rivaroxaban group, the AUEC of FXa inhibition showed no significant change after R-CHOP (mean difference 3.8 ng·h/mL, 95% confidence interval (CI) −155.4 to 163.0, *p* = 0.96). Similarly, in the dabigatran group, the AUEC of dTT remained unchanged post R-CHOP (mean difference 54.41 ng·h/mL, 95% CI −99.09 to 207.9 ng/mL, *p* = 0.46). However, the median time-to-peak dTT was significantly faster with R-CHOP (3 h, [min–max, 1.5–8] compared to without it (4 h, [min–max, 3–8], *p* = 0.04); **Conclusions**: Concurrent R-CHOP chemotherapy did not significantly impact FXa inhibition by rivaroxaban or dTT by dabigatran. The time-to-peak dTT was faster when dabigatran was administered with R-CHOP.

## 1. Introduction

Venous thromboembolism (VTE) is a common complication of malignancy and a presenting feature of an occult malignancy. The risk of VTE in cancer patients is 4- to 7-fold higher than in the general population [1]. Moreover, the incidence of fatal bleeding is higher among patients who are concurrently diagnosed with cancer-associated thrombosis (CAT) when compared to patients without active cancer [2]. Patients with cancer who develop VTE have a reduced life expectancy compared to those without [1,3,4,5].

The diagnosis of VTE has been more commonly reported in ambulatory care than inpatient care [6,7]. The risk of VTE is further increased with chemotherapy [8]. Both arterial thrombosis and VTE are among the leading causes of death in patients who receive chemotherapy [9,10].

The advent of direct oral anticoagulants (DOAC) in the 2000s significantly changed the perspective of anticoagulation and rapidly replaced classical anticoagulants. Recent data from the real-world GARFIELD-VTE registry revealed an increase in DOAC use in clinical practice [11]. Although DOAC have fewer drug–drug interactions (DDI) than vitamin K antagonists, drugs that affect drug transporters (e.g., P-gp) and/or common metabolic enzymes (e.g., CYP3A4) may affect the plasma concentrations of DOAC and lead to clinically significant alterations in their anticoagulant effects and consequently a higher risk of thromboembolic events or bleeding.

Combination chemo-immunotherapy with rituximab or R-CHOP (rituximab, cyclophosphamide, doxorubicin, vincristine, and prednisone) remains the standard of care for previously untreated diffuse large B-cell lymphoma (DLBCL). All the chemotherapeutic agents within the R-CHOP regimen, except rituximab, may potentially interact with co-administered DOAC. Vincristine is a substrate of CYP3A4 and P-gp and also an inhibitor of CYP3A4. Cyclophosphamide is a substrate and inhibitor of CYP3A4. Doxorubicin is a substrate and inhibitor of CYP3A4, and it is also an inducer of P-gp. Prednisone is a substrate and inducer of CYP3A4 (Table S1).

Several pivotal randomized controlled trials have demonstrated that DOAC can be efficaciously and safely used in patients with active cancer compared to standard low-molecular-weight heparin treatment [12,13,14]. However, guidance from the Scientific and Standardization Committee of the International Society on Thrombosis and Haemostasis (SCC of the ISTH) states that DOAC should be cautiously used in patients receiving CYP3A4 or P-gp inhibitors/inducers [15]. DDI are difficult to predict with a combination chemotherapy regimen and DOAC with potentially multiple interactions. Indeed, the ‘net’ DDI of the co-administration of common chemotherapy regimens and DOAC plasma concentrations are often unknown.

In this study, we investigated the impact of the R-CHOP combination chemo-immunotherapy regimen on the pharmacodynamics of two DOAC, rivaroxaban and dabigatran, in adults with non-Hodgkin lymphoma.

## 2. Materials and Methods

### 2.1. Study Population

This was a prospective, open-label, randomized, single-center pharmacodynamic study, enrolling consecutive DLBCL patients who were scheduled to receive R-CHOP chemo-immunotherapy. Eligible participants were 18 years or older and had a body mass index between 18.5 and 29.9 kg/m^2^ and a Khorana score of 2 or higher. None of the patients had a central nervous system involvement of lymphoma. Subjects were excluded if they had significant liver or renal disease or dysfunction defined as aspartate transaminase (AST) or alanine aminotransferase (ALT) more than three times the upper limit of normal (ULN), total bilirubin more than two times the ULN, and a calculated creatinine clearance of less than 30 mL per minute (calculated by the Cockcroft–Gault formula). Participants also had no history of clinically significant major bleeding. Other exclusion criteria included concurrently treated with anticoagulants or antiplatelets and pregnancy or lactating. Full eligibility criteria are provided in the Data Supplement.

All participants provided written informed consent. The study protocol was approved by the Research Ethics Committee of the Faculty of Medicine, Chiang Mai University (MED-2562-06700).

### 2.2. Sample Size

To demonstrate the pharmacodynamic differences in dabigatran, the sample size was based on ensuring precision in parameter estimates. Based on the estimated difference of 50% of the mean area under the effect curve (AUEC) and a standard deviation of 242 and assuming average AUEC results are normally distributed, a sample size of 12 subjects is at least required.

To demonstrate the difference in the pharmacodynamics of rivaroxaban accurately, the sample size was based on the ensuring precision in parameter estimates. Based on the estimated difference of 50% of the mean AUEC and a standard deviation of 800, and assuming average AUEC results are normally distributed, a sample size of 12 subjects is at least required.

Therefore, we planned to enroll a total of 26 subjects, which included 13 subjects for the dabigatran group and 13 subjects for the rivaroxaban group.

### 2.3. Study Medication

Participants received rivaroxaban 10 mg orally with a meal or dabigatran etixalate 110 mg orally every 12 h.

### 2.4. Blood Sampling for Pharmacodynamic Assessment

**Figure 1:**
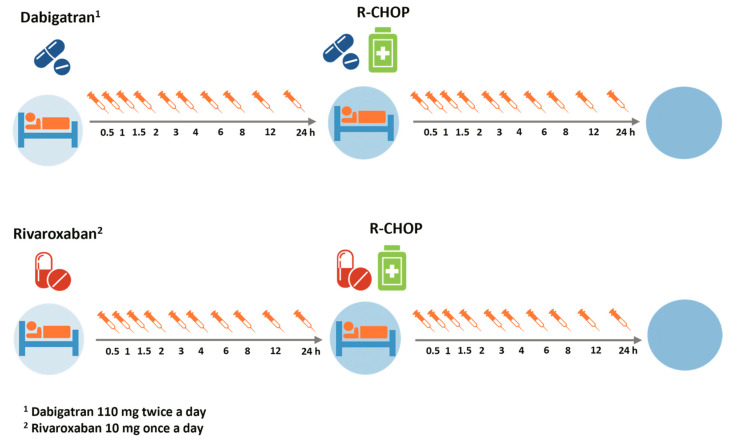
Schedule sample collection.

Blood samples were obtained to assess the pharmacodynamics of rivaroxaban and dabigatran on two separate occasions: the first timepoint was the day before starting R-CHOP chemotherapy and the second timepoint following the initiation of R-CHOP chemotherapy. The time interval between the two occasions was 24 h. Blood samples were drawn immediately before the administration of the DOAC and at 0.5, 1, 1.5, 2, 3, 4, 6, 8, 12 and 24 h post-DOAC intake (Figure 1). Plasma anti-Xa activity was measured for participants in the rivaroxaban group and plasma diluted thrombin time (dTT) for participants in the dabigatran group [16,17,18].

After the completion of the sample collection, all participants were monitored for bleeding symptoms for 42 days.

### 2.5. Measurement of Anti-Xa and Diluted Thrombin Time

Anti-Xa activity was measured by a modified chromogenic method using Biophen^TM^ Heparin reagent (Hyphen BioMed, Neuville-sur-Oise, France). This method is a one-stage chromogenic assay based on the inhibition of a constant amount and excess of FXa [19]. The residual FXa hydrolyses a specific chromogenic substrate (SXa-11) releasing paranitroaniline (pNA), which is quantified by spectrophotometry. Two milliliters of blood were collected from each participant (3.2% sodium citrate tube), centrifuged within 30 min and the plasma tested using an automated coagulation analyzer, SYSMEX CS-2500 (Sysmex Corporation, Kobe, Japan). The assay was calibrated to detect anti-Xa activity within a range of 0–454 ng/mL based on the manufacturer’s recommendations and was validated in the laboratory prior to the study.

Diluted thrombin time (dTT) was measured using a HemosIL Direct Thrombin Inhibitor (DTI) assay (Instrumentation Laboratory Company, Bedford, MA, USA) [20]. The assay was calibrated to detect dTT within a range of 0–470 ng/mL, following the manufacturer’s recommendations, and was also validated in the laboratory prior to conducting the study. Plasma samples were processed and tested using the automated coagulation analyzer, ACL TOP Family (Instrumentation Laboratory Company, Bedford, MA, USA), and compared to the reference value.

### 2.6. Statistical Analysis

Pharmacodynamic parameters, including anti-FXa and dTT were determined using a non-compartmental analysis (PKSolver software) [21]. The parameters included AUEC of FXa inhibition and dTT over the specified time intervals (AUEC_0–12_ and AUEC_0–24_) as well as the maximum FXa inhibition or dTT. Both the arithmetic mean with corresponding standard deviation (SD) and geometric mean were reported for these parameters. The differences in means of AUEC were compared using mixed-effects model. The coefficient of variation (CV%) was also calculated for each group to assess variability in the data. Time-to-peak FXa inhibition or dTT was defined as the time to reach the maximum FXa inhibition or dTT after administration. The difference in the time-to-peak maximum FXa inhibition or dTT was compared using a *t*-test or Wilcoxon Rank Sum test, as appropriate. A *p*-value of less than 0.05 was considered statistically significant. Non-pharmacodynamic statistical analyses were performed using Stata (version 16; StataCorp, College Station, TX, USA). Descriptive statistics were used for the analysis of all other parameters.

## 3. Results

A total of 26 participants were enrolled, with 12 receiving rivaroxaban and 14 receiving dabigatran. No participants withdrew. Baseline characteristics are shown in Table 1. The mean age (±SD) was 59 years (±14.4) with six (50%) and eight (57%) males in the rivaroxaban and dabigatran groups, respectively. In both groups, the median Khorana score was 2, which accounted for 85% of the study population. One participant in each group had a Khorana score of 3. Only one participant in the rivaroxaban group had a Khorana score greater than 4.

In the rivaroxaban group, the mean body weight (±SD) was 49.1 kg (±7.1), height (±SD) 155.5 cm (±7.2), and BMI (±SD) 20.3 kg/m^2^ (±2.6), while in the dabigatran group, the body weight was 56.0 kg (±9.0), height 159.1 cm (±5.8), and BMI 22.1 kg/m^2^ (±3.4).

### Outcomes

**Figure 2:**
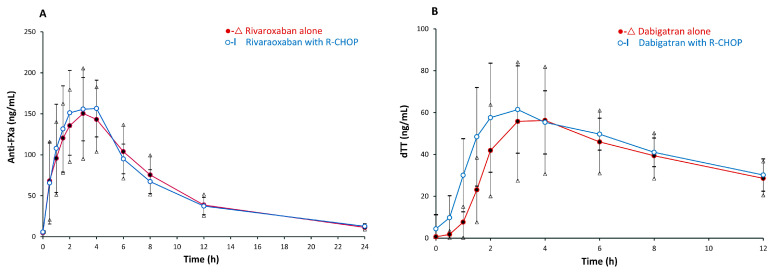
Mean concentrations versus time curves (±95% confidence interval) in patients taking (**A**) rivaroxaban and (**B**) dabigatran alone and in combination with R-CHOP. R, rituximab; C, cyclophosphamide; H, doxorubicin; O, vincristine; P, prednisolone.

Table 2 summarizes the pharmacologic parameters of rivaroxaban and dabigatran. The arithmetic mean ± SD of AUEC_0–24_ was 1422.0 ± 457.1 ng·h/mL when rivaroxaban was administered alone and 1425.9 ± 375.1 ng·h/mL when rivaroxaban was co-administered with R-CHOP, with a mean of differences 3.8 ng·h/mL (95% confidence interval (CI) −155.4 to 163.0, *p* = 0.96), Figure 2A and Supplementary Figure S1. The geometric mean AUEC_0–24_ was 1354.0 ng·h/mL when rivaroxaban was administered alone and 1384.2 ng·h/mL when co-administered with R-CHOP, (*p* = 0.85). The arithmetic mean AUEC_0–∞_ was 1565.0 ± 485.2 ng·h/mL when rivaroxaban was administered alone and 1576.6 ± 415.4 ng·h/mL when co-administered with R-CHOP. The maximum inhibition of FXa was 187.0 ± 75.7 ng/mL compared to 196.5 ± 61.5 ng/mL, (*p* = 0.55) when comparing adults taking rivaroxaban without and with R-CHOP, as shown in Table 2. To further assess the interaction, a bioequivalence analysis was performed. For the AUEC, the geometric mean ratio between rivaroxaban alone and rivaroxaban with R-CHOP was 0.98 (90% CI 0.90 to 1.07). For the maximum inhibition of FXa, the geometric mean ratio was 0.92 (90% CI 0.79 to 1.08).

The arithmetic mean AUEC_0–12_ was 449.2 ± 275.3 ng·h/mL and 503.6 ± 187.2 ng·h/mL when dabigatran was administered alone and with R-CHOP, respectively. The mean difference in AUEC_0–12_ was 54.41 ng·h/mL (95% CI −99.09 to 207.9 ng/mL, *p* = 0.46), Figure 2B and Supplementary Figure S1. The geometric mean AUEC_0–12_ was 350.6 ng·h/mL when dabigatran was administered alone and 476.2 ng·h/mL when co-administered with R-CHOP (*p* = 0.18). The arithmetic mean AUEC_0–∞_ was 791.7 ± 603.6 ng·h/mL when dabigatran was administered alone and 1703.3 ± 2250.1 ng·h/mL when dabigatran was co-administered with R-CHOP. After excluding outliers, the recalculated mean AUEC_0–∞_ for the dabigatran + R-CHOP group was 940.6 ± 618.0 ng·h/mL. The geometric mean ratio for the AUEC and Cmax were determined to assess bioequivalence. For the AUEC, the geometric mean ratio between dabigatran alone and dabigatran with R-CHOP was 1.89 (90% CI 1.06 to 3.35). For maximum dTT, the geometric mean ratio was 1.32 (90% CI 0.87 to 2.01). The maximum dTT was 75.8 ± 34.1 ng/mL compared to 76.2 ± 30.9 ng/mL (*p* = 0.95) when comparing dabigatran without and with R-CHOP, as shown in Table 2. As the second dose of dabigatran was administered at another 12 h interval, it was expected that the AUEC would increase once more. Nonetheless, there is no statistically significant difference in AUEC_0–24_.

The median time-to-maximum FXa inhibition for rivaroxaban exhibited no statistically significant difference when administering with or without R-CHOP, with medians of 1.75 h (min–max, 1–4 h) and 2.5 h (min–max, 0.5–6 h), respectively (*p* = 0.78).

However, in the case of dabigatran, the median time-to-peak dTT was significantly faster in patients receiving dabigatran concurrently with R-CHOP (median 3 h, [min–max, 1.5–8 h]) compared to when it was administered without it (median 4 h, [min–max, 3–8 h]), *p* = 0.04.

There were no adverse events reported in either group and no participants had clinically relevant non-major bleeding or major bleeding over the 42-day follow-up period.

## 4. Discussion

To the best of our knowledge, this is the first study assessing the impact of combined chemotherapy on rivaroxaban and dabigatran in adult cancer patients. We demonstrated that the R-CHOP regimen did not significantly alter the pharmacodynamics of rivaroxaban and dabigatran. No significant change in FXa inhibition or dTT was observed when DOAC were co-administrated with R-CHOP. Although the peak FXa inhibition of rivaroxaban and dTT of dabigatran slightly increased with R-CHOP, these changes were not statistically significantly different. Of note, patients receiving dabigatran with R-CHOP exhibited a faster median time-to-peak dTT compared to those without R-CHOP. Among the 26 participants, no clinically significant bleeding related to DOAC administration was observed.

Given the common drug transporter and metabolic enzyme pathways used by drugs in R-CHOP and both rivaroxaban and dabigatran, we hypothesized that the pharmacodynamic effect of rivaroxaban and/or dabigatran may be influenced when used in combination with R-CHOP. If the effect of rivaroxaban on the inhibition of factor Xa increased, decreased, or remained unchanged, we anticipated that the effect on dabigatran’s dTT would not follow the same trend. This hypothesis was based on the understanding that rivaroxaban is a substrate of CYP3A4 and P-gp, while dabigatran is only a substrate of P-gp. Our results indicate that any alterations in CYP3A4 and P-gp were not large enough to alter the pharmacodynamic effect of dabigatran or rivaroxaban.

Further analysis using a bioequivalence approach for rivaroxaban revealed no meaningful difference in overall exposure when rivaroxaban was co-administered with R-CHOP. In contrast, the bioequivalence analysis for dabigatran indicated a modest increase in systemic exposure and peak concentrations when co-administered with R-CHOP. This was supported by a mixed-effects model analysis, which showed significant interactions at the 1 h and 1.5 h timepoints for dabigatran, suggesting that the absorption of dabigatran may be slightly accelerated when administered with R-CHOP. The faster rise in dTT further supports this hypothesis, potentially due to the modulation of P-gp activity or other pathways. Nonetheless, it is important to note that no significant interactions were identified when comparing the administration of dabigatran alone versus with R-CHOP.

Dabigatran is formulated as a capsule containing tartaric acid pellets [22,23]. These pellets create an acidic environment and enhance dabigatran absorption independently of gastric pH. It is important to highlight that the R-CHOP in our regimen study included omeprazole to alleviate gastrointestinal symptoms from high-dose corticosteroids. It is known that dabigatran plasma concentrations can decrease when co-administrated with proton pump inhibitors (PPIs) [23,24,25]. Similar results were reported in the RE-LY trial, which showed that the co-administration of PPIs decreased the area under the plasma concentration time curve by 12.5%, suggesting that the bioavailability of dabigatran may be decreased when co-administrated with PPIs [24]. Nevertheless, our study was not designed to directly compare the outcomes of these two groups. While we observed no statistically significant changes in AUEC, we noted that the concentration curve of dTT exhibited a faster rise following the administration of dabigatran with chemotherapy. This finding reinforces our hypothesis regarding the potential acceleration of dabigatran absorption when co-administered with R-CHOP.

There was a variation in FXa inhibition and dTT after the administration of a DOAC with and without R-CHOP. This variance could potentially be attributed for to intra- and interindividual differences in drug concentrations. In a recent study led by Toorop et al. [25], the evaluation of DOAC trough and peak concentrations at three different timepoints revealed intra-individual variability, with a coefficient of variation ranging between 18% and 33%. Our study exclusively included patients diagnosed with DLBCL and treated with the R-CHOP immunochemotherapy regimen to ensure control over variations in disease and treatment. Although there were slight differences in the individual baseline characteristics among patients, we mitigated concerns by using a self-control method to determine the difference in drug concentration before and after chemotherapy.

Despite observing variations in drug concentration before and after chemotherapy, our analysis did not show any significant differences.

Current ISTH and ASCO guidelines recommend that DOAC can be used for CAT treatment and mention that high-risk outpatients with cancer (with a Khorana score of 2 or higher) may be offered thromboprophylaxis including DOAC [26,27]. Both guidelines place importance on the risk of DDI. Our study provides reassuring data that rivaroxaban and dabigatran have a low potential of DDI with R-CHOP for adults with previously untreated DLBCL.

The European Heart Rhythm Association (EHRA) listed potential chemotherapy and DOAC interactions in the most recent DOAC guidance [28]. This guideline recommends against the use of doxorubicin together with a DOAC due to the concern of reduced DOAC drug concentrations. It is important to note that most of the recommendations are based on the pharmacokinetics data of DOACs and chemotherapy. However, most of the interactions were not examined in human subjects. Therefore, the recommendations of DDI and DOACs should be revisited.

Our study has some limitations. First, we did not measure the actual DOAC plasma concentrations; however, studies have reported a good correlation between anti-Xa activity and plasma rivaroxaban concentration, as well as dTT and plasma dabigatran concentration [16,17,18]. Secondly, we collected blood samples after the administration of the first dose of rivaroxaban and dabigatran because of safety concerns for the participants. The drug concentration will not reflect the effect at steady-state plasma concentrations. Finally, our study had a relatively short follow-up and was not designed to explore the clinical relevance, such as long-term thrombosis events.

## 5. Conclusions

In conclusion, concomitant administration of R-CHOP chemotherapy showed no significant impact on rivaroxaban or dabigatran pharmacodynamics. However, time-to-peak dTT was faster when dabigatran was administered with R-CHOP.

## Figures and Tables

**Table 1:** Characteristics of participants.

Baseline Characteristics	Total (*n* = 26)	Group of DOAC Administration (*n* = 26)	
		Rivaroxaban 10 mg (*n* = 12)	Dabigatran 110 mg (*n* = 14)
Sex, *n* (%)			
Male	14 (53.9)	6 (50.00)	8 (57.14)
Age—mean ± SD	59.4 ± 14.4	61.8 ± 14.9	57.3 ± 14.1
Weight (kg)—mean ± SD	52.9 ± 8.8	49.1 ± 7.1	56.0 ± 9.0
Height (cm)—mean ± SD	157.5 ± 6.6	155.5 ± 7.2	159.1 ± 5.8
BMI (kg/m^2^)—mean ± SD	21.30 ± 3.1	20.3 ± 2.6	22.1 ± 3.4
BSA—mean ± SD	1.5 ± 0.1	1.5 ± 0.1	1.6 ± 0.1
Ann Arbor stage, n (%)			
I	2 (7.7)	0 (0)	2 (14.3)
II	8 (30.8)	3 (25.0)	5 (35.7)
III	4 (15.4)	2 (16.7)	2 (14.3)
IV	12 (46.1)	7 (58.3)	5 (35.7)
Khorana score, *n* (%)			
≤2	23 (88.5)	10 (83.3)	13 (92.9)
>2	3 (11.5)	2 (16.7)	1 (7.1)
Medical history, *n* (%)			
Hypertension	5 (19)	3 (25)	2 (14.3)
Diabetes mellitus	1 (4)	0 (0)	1 (7.1)
Dyslipidemia	1 (4)	0 (0)	1 (7.1)
Concomitant medications, *n* (%)			
Calcium channel blockers	3 (11.5)	1 (8.3)	2 (14.3)
ACE inhibitors	1(3.8)	1 (8.3)	0 (0.0)
Diuretic	1(3.8)	1 (8.3)	0 (0.0)
Statin	1(3.8)	0 (0)	1 (7.1)
Metformin	1 (3.8)	0 (0.0)	1 (7.1)
Sulfonylureas	1 (3.8)	0 (0.0)	1 (7.1)
Laboratory—mean ± SD			
Hemoglobin (g/dL)	10.9 ± 1.5	10.5 ± 1.7	11.2 ± 1.4
WBC (10^9^/L)	7192.3 ± 4954.2	7515.0 ± 4496.0	6915.7 ± 5469.6
Platelet (10^9^/L)	338.5 ± 151.9	372.7 ± 201.0	309.1 ± 90.4
Creatinine clearance (ml/min)	68.9 ± 21.8	61.1 ± 22.3	75.7 ± 19.7
LDH (U/L) *	240.4 ± 69.5	241.8 ± 64.4	239.1 ± 76.5
Uric (mg/dL)	5.6 ± 1.2	5.5 ± 1.4	5.7 ± 1.1
Albumin (g/dL)	4.1 ± 0.3	4.0 ± 0.3	4.2 ± 0.2
Aspartate aminotransferase (U/L)	26.1 ± 10.3	29.5 ± 13.0	23.2 ± 6.7
Alanine aminotransferase (U/L)	26.0 ± 18.1	32.5 ± 24.5	20.5 ± 6.9
Alkaline phosphatase (U/L)	115.1 ± 116.2	90.8 ± 35.9	136.0 ± 154.5
Total bilirubin (mg/dL)	0.3 ± 0.2	0.3 ± 0.2	0.4 ± 0.2
Direct bilirubin (mg/dL)	0.7 ± 2.7	0.2 ± 0.1	1.1 ± 3.7

ACE inhibitors: angiotensin-converting enzyme inhibitors; BMI: body mass index; BSA: body surface area; DOAC: direct oral anticoagulants; LDH: lactate dehydrogenase; WBC: white blood count. * LDH data are missing in one patient in the dabigatran group.

**Table 2:** Pharmacologic parameters of rivaroxaban and dabigatran predicted by the concentrations of FXa and dTT when administered alone and in combination with R-CHOP.

Parameter	DOAC Alone		DOAC with R-CHOP		*p* Value
	Mean ± SD	CV (%)	Mean ± SD	CV (%)	
Rivaroxaban					
Arithmetic AUEC_0–24_ (ng·h/mL)	1422.0 ± 457.1	32	1425.9 ± 375.1	26	0.96
Arithmetic AUEC_0–∞_ (ng·h/mL)	1565.0 ± 485.2	31	1576.6± 415.4	26	0.88
Geometric AUEC_0–24_ (ng·h/mL)	1354.0	33	1384.2	25	0.85
Geometric AUEC₀_–∞_(ng·h/mL)	1464.7	36	1520.9	27	0.67
Maximum inhibition of FXa (ng/mL)	187.0 ± 75.7	40	196.5 ± 61.5	31	0.55
E_max_ (ng/mL)	138.87 ± 75.7	42	165.79 ± 91.87	33	0.55
Time to maximum inhibition of FXa (h) *	2.5 (0.5–6)	60	1.75 (1.0–4.0)	50	0.78
Dabigatran etixalate					
Arithmetic AUEC_0–12_ (ng·h/mL)	449.2 ± 275.3	61	503.6 ± 187.2	37	0.46
Arithmetic AUEC₀_–∞_(ng·h/mL)	791.7 ± 603.6	76	1703.3 ± 2250.1	132	0.16
Geometric AUEC_0–12_ (ng·h/mL)	350.6	80	476.2	42	0.18
Geometric AUEC₀_–∞_(ng·h/mL)	552.27	109	1042.71	216	0.16
Maximum dTT (ng/mL)	75.8 ± 34.1	45	76.2 ± 30.9	41	0.95
E_max_ (ng/mL)	67 ± 42.8	42	74 ± 35	32	0.56
Time to maximum dTT (h) *	4.0 (3.0–8.0)	64	3.0 (1.5–8)	47	0.04

AUEC, area under the effect–time curve; CV, coefficient of variation; DOAC, direct oral anticoagulants; E_max_, maximum effect; SD, standard deviation; R-CHOP, rituximab, cyclophosphamide, doxorubicin, vincristine, prednisolone. * Median (range).

## Data Availability

The original contributions presented in this study are included in the article/Supplementary Material; further inquiries can be directed to the corresponding author.

## Supplementary Materials

The following supporting information can be downloaded at https://www.mdpi.com/article/10.3390/pharmaceutics16101319/s1, Figure S1: Mean area under the effect curve (AUC) of rivaroxaban and dabigatran level, comparing the administration DOAC alone and in combination with R-CHOP; Table S1: R-CHOP with CYP3A4 and P-glycoprotein interactions.
