# Supplementary material for: Pharmacodynamics of Rivaroxaban and Dabigatran in Adults with Diffuse Large B-Cell Lymphoma Receiving R-CHOP Immunochemotherapy
Source: Pharmaceutics. 2024 Oct 11;16(10):1319. doi: 10.3390/pharmaceutics16101319 (PMC11511075; doi:10.3390/pharmaceutics16101319)
Supplement: Supplementary file 1 [file pharmaceutics-16-01319-s001.zip › pharmaceutics-3191656-supplementary.pdf]

Supplementary Materials

Full Inclusion and Exclusion Criteria

Inclusion Criteria

Participants were enrolled with:

- Informed consent
- Age ≥ 18 years
- Body mass index (BMI) of 18.5-29.9 kg/m^2
- Newly diagnosed non-Hodgkin lymphoma, diffuse large B-cell lymphoma subtype, planned to be treated with R-CHOP chemotherapy regimen
- Khorana score of 2 or higher

Exclusion Criteria

If a patient meets any of the following criteria, he or she was not eligible:

- Significant liver disease or dysfunction (AST or ALT > 3 times ULN, Total bilirubin greater than > 2 times ULN). Examples of significant liver disease including acute clinical hepatitis, chronic active hepatitis, or cirrhosis
- Severe renal impairment, defined as calculated creatinine clearance (CrCl) less than 30 mL/min
- Significant respiratory, cardiovascular, metabolic, endocrine, or neurologic disorders
- Uncorrected mucosal lesions
- Central nervous system involvement of lymphoma
- High disease burden, tumor lysis syndrome, or require emergency radiation therapy

- Poor performance status, evaluated by the Eastern Cooperative Oncology Group (ECOG) performance status equal or greater than 3
- Recent surgery within 30 days of the enrollment
- Recently major bleeding within 90 days of the enrollment
- History of intracranial bleeding
- Concurrent treatment with anticoagulants (LMWH, DOAC, or warfarin) or antiplatelets
- Pregnant or lactating

Abbreviations: ALT, alanine aminotransferase; AST, aspartate aminotransferase; DOAC, direct oral anticoagulants, LMWH: low molecular weight heparin, ULN: upper limit of normal; R-CHOP, rituximab, cyclophosphamide, doxorubicin, vincristine, and prednisone

Figure S1. Mean area under the effect curve (AUC) of rivaroxaban and dabigatran level comparing between administration DOAC alone and in combination with R-CHOP.

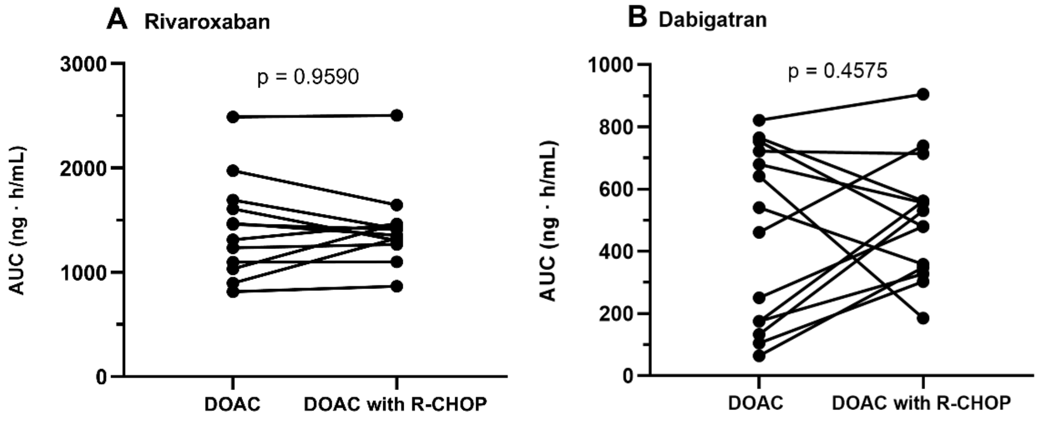

Table S1. R-CHOP with CYP3A4 and P-glycoprotein interactions.

	**CYP3A4 Interactions** [a]			**P-Glycoprotein Interactions** [b,c]		
	**Substrate**	**Inducer**	**Inhibitor**	**Substrate**	**Inducer**	**Inhibitor**
Rituximab						
Cyclophosphamide	+		+			
Doxorubicin	+++		+	•	•	
Vincristine	+++		+	•		
Prednisone	+	++				

Adapted from Short NJ, Connors JM: New oral anticoagulants and the cancer patient. Oncologist 2014 Jan;19(1):82-93. doi:10.1634/theoncologist.2013-0239. PMID: 24319019.

[a] +++, strong interaction; ++, moderate interaction; +, weak interaction;

[b] Data for the strength of P-glycoprotein interactions are limited. •, indicates that interaction has been documented;

[c] Bold indicated the drug is either a strong or a moderate inhibitor of CYP3A4 (with or without P-glycoprotein interaction). These drugs have an increased risk for interactions with the new oral anticoagulants that are metabolized by CYP3A4.
